# Circulating Elabela-21 and Elabela-11 levels as predictive biomarkers in individuals with recently developed type 2 diabetes

**DOI:** 10.4314/ahs.v25i3.18

**Published:** 2025-09

**Authors:** Abdullah Abbas Hamzah Al-Rubaye, Walaa Esmail Jasim, Ahmed A Mohsin

**Affiliations:** 1 Department of Medical Laboratory Technology, College of Health and Medical Technology, Middle Technical University, Baghdad, Iraq; 2 Department of Medical Laboratory Technology, College of Health and Medical Technology, Southern Technical University, Basra, Iraq

**Keywords:** Lipid profile, Prediabetes, Elabela, Glycated hemoglobin, Glucose

## Abstract

**Background:**

Type 2 diabetes mellitus (T2DM) is significantly influenced by the Elabela hormone, Elabela has been discovered to be a new endogenous apelin receptor (APJ) ligand.

**Objective:**

To detect the potential changes of Elabela-21 and Elabela-11 levels in recently developed T2DM.

**Materials and Methods:**

A recent cross-sectional study with 180 participants was conducted. The participants were divided into three groups based on WHO classifications: T2DM patients, prediabetic cases, and healthy controls. Five milliliters of blood samples were collected in the morning during 9-12 hours of fasting. Tests were carried out for each patient and control involving glucose and lipid profile using the enzymatic methods by a biochemical auto-analyzer, glycated hemoglobin using the ion exchange high-performance liquid chromatography (HPLC), Elabela-21, and Elabela-11 using an ELISA technique.

**Results:**

Serum Elabela-21 level in T2DM patients was (803.2067 ± 11.47459 pg/mL), and in prediabetic cases, it was (641.2417 ± 3.216256 pg/mL), which was higher than the value of the healthy controls (542.6983 ± 3.59739 pg/mL), and the differences were statistically significant (P < 0.001). Also, serum Elabela-11 level was significantly higher in people with T2DM and prediabetic cases (502.6683 ± 11.51197 and 339.25 ± 3.452898 pg/mL, P < 0.001), respectively, compared to the healthy controls (140.83 ± 3.603656 pg/mL). These differences were statistically significant (P < 0.001).

**Conclusion:**

Elabela-21 and Elabela-11 levels in the blood gradually increased from prediabetes to recently developed T2DM. They were positively correlated with body mass index, glycemic, and lipid profile and negatively associated with HDL.

## Introduction

Type 2 diabetes mellitus (T2DM) is a highly prevalent form of metabolic disease that spreads throughout the world and is developed by two primary contributors: either inadequate insulin production or an inability of insulin-sensitive tissues to respond in the right way to insulin^1^. Pancreatic beta-cell efficiency and insulin significant play major roles in the pathophysiology of T2DM^2^, ^3^. Polyuria, polydipsia, and unintentional weight loss are the common symptoms of T2DM^4^, ^5^. While a large number of patients with recently developed T2DM is asymptomatic and identified by screening tests such as fasting plasma glucose or hemoglobin A1c (HbA1c)^6^.

The apelin system, which is composed of the apelin receptor (APJ) and its two endogenous peptide ligands, apelin and Elabela, are endogenous physiological mediators in many types of conditions, including T2DM^7^.

Elabela, a hormone peptide that belongs to the adipokines, significantly maintains glucose levels, lipid metabolism, and numerous other physiological functions in the body, making it crucial for both the prevention and management of metabolic illnesses like T2DM^8^.

The hormone-related peptide that serves as the second endogenous ligand used by the G-protein-coupled APJ has the name Elabela (ELA), which is also referred to as Toddler or Apela. ELA is abundantly generated in human embryonic, cardiac, and renal tissues. It has a vital role in several biological processes, including preserving bodily fluid homeostasis, regulating blood circulation, and developing embryos^9^.

The 54-amino-acid pre-ELA protein that the ELA gene produces contains a signal peptide at its N-terminus. Following translocation into the endoplasmic reticulum (ER) and signal peptide splitting, the 32-amino-acid pro-ELA protein can produce multiple functional fragments. Little molecular isoforms like ELA-21 and ELA-11 are generated when ELA-32 is broken down by the Golgi apparatus and ER^10^. The paraventricular nucleus (PVN) of the central nervous system contains APJ. ELA-21 reduced food consumption by stimulating neurons in the PVN that produce antidiuretic hormone (ADH) and corticotropin-releasing hormone in the hypothalamus. Thus, ELA is classified as a hormone that operates on the brain to decrease appetite^11^.

In the kidney, ELA is more strongly generated. Therefore, it can increase water consumption and diuresis to maintain fluid equilibrium^12^. The present study was designed to identify the levels of ELA-21 and ELA-11 in prediabetes and recently developed T2DM that may help in the early diagnosis of the disease.

## Subjects and Methods

### Subjects

This study involved 180 subjects, categorized into three groups: a healthy control, prediabetes, and T2DM. For each group, there were sixty participants. Prediabetes and T2DM groups were newly diagnosed with the disease and attended specialized clinics at Al-Fayhaa Teaching Hospital in Basra City. Their ages ranged from 30 to 75 years. The diagnosis of cases with prediabetes and T2DM was based on WHO criteria by fasting serum glucose and HbA1c^13^. Healthy controls were not diabetic and non-smokers. Patients who were taking drugs affecting glucose metabolism, pregnant women, and kidney, cardiovascular, liver, or thyroid gland disorders were excluded from this study.

### Blood collection

A disposable butterfly needle was used to collect 5 milliliters (mL) of venous blood from every participant in the morning during 9-12 hours of fasting which was then divided into two tubes.

First, 2 mLs of blood were transferred into an EDTA K3-containing vacutainer tube for the immediate estimation of HbA1c.

Second, 3 mLs of blood were transferred into a gel and clot activator-containing evacuated tube and stood for 30 minutes, then centrifuged for 15 minutes at 3000 revolutions per minute (RPM) for the separation of serum to estimate glucose and lipid profile levels immediately. The remaining serum was transferred into an Eppendorf tube and frozen in deep freeze at -80°C for subsequent analysis of ELA-21 and ELA-11.

Blood samples were collected during the period from the beginning of April 2024 to the end of August 2024. The information such as name, age, sex, height, and weight were collected from all participant. Tests were carried out for each patient and control involving glucose, lipid profile (total cholesterol (TC), triglycerides (TG), high density lipoprotein (HDL), low density lipoprotein (LDL), and very low-density lipoprotein (VLDL)), and HbA1c in Al-Amal Medical Laboratory as well as, ELA-21 and ELA-11 in the Biological Technologies Laboratory in the College of Health and Medical Technology/Basra.

## Methods

### Anthropometry

After using a stadiometer to determine standing height and a balance to measure weight, the body mass index (BMI) was computed using the formula below:

BMI = Weight (Kilogram)/Height (meter^2^)^14^.

### Biochemical measurements

The enzymatic (glucose oxidase) method assessed serum glucose using a biochemical autoanalyzer (Spin 200 analyzer, Spinreact Company, Spain).

Cholesterol oxidase, peroxidase, and the chromogen 4-aminophenazone/phenol were utilized in an enzymatic procedure to evaluate serum TC and HDL-C^15^ instantly in a full automation biochemistry analyzer (Spin 200 analyzer, Spinreact Company, Spain).

An enzymatic technique employing lipoprotein lipase, glycerokinase, glycerophosphate oxidase, and the chromogen 4-aminophenazone/N-ethyl-N (3-sulphopropyl)-nramisidine allowed for the instant detection of serum TG concentration^16^ in a fully automated biochemistry analyzer (Spin 200 analyzer, Spinreact Company, Spain). LDLL–C and VLDL were calculated using Friedwald's formula^17^.

### Hormones measurements

The ion exchange high-performance liquid chromatography (HPLC) technique in a full automation analyzer (D-10 analyzer, Bio-Rad Company, USA) was used in the detection of HbA1c in whole blood.

The levels of fasting serum ELA-21 and ELA-11 were identified by centrifuging the frozen serum for 5 minutes at 3000 RPM after they had been thawed for 30 minutes at ambient temperature (20–25°C). They were estimated by applying quantitative sandwich-based enzyme-linked immunosorbent assay (ELISA) kits (LOT No. 202406, Shanghai Ideal Medical Technology Company, China). A full automation system was used with the Elisys Uno (ELISA) instrument (Human company, Germany).

### Statistical analysis

SPSS version 26.0 was employed to complete all data analysis. For group comparison, either an unpaired or paired t-test was applied. The relationship analysis between numerical parameters was examined using Pearson's correlation coefficients. The sensitivity and specificity of ELA-21 and ELA-11 for prediabetes and T2DM were given by receiver operating characteristic (ROC) curves. The data's mean and standard errors (SE) were displayed in contrast to healthy controls. *P* value less than 0.05 was considered statistically significant.

### Ethics-based permission

The Middle Technical University's Medical Ethics Committee (MEC number 063 on January 1, 2024) at Al Za'franiya, Baghdad Governorate, awarded ethical approval for this study. The subjects gave written consent to participate in this study. This study was carried out in full accordance with the Helsinki Declaration.

## Results

A total of 180 participants, T2DM, prediabetes, and age-, sex-, and BMI-matched healthy controls, were included in the present study, as shown in the baseline characteristics in Table 1. Compared to the healthy controls, T2DM patients and prediabetic cases had significantly higher glucose and HbA1c. Moreover, lipids, including fasting serum TC, TG, VLDL, and LDL-C in T2DM patients and prediabetic cases, were higher than in healthy controls, in contrast to controls, there was a lower HDL-C level in T2DM and prediabetic cases (P < 0.001).

**Table 1:** The anthropometric and clinical characteristics for the participants

Parameters	Study groups	Mean	Standard Error (SE)	P-Value
Age (years)	Control (n=60)	51.8	1.748898	>0.05
	Prediabetes (n=60)	52.75	1.665981	>0.05
	T2DM (n=60)	52.48333	1.508449	>0.05
BMI (kg/m^2^)	Control (n=60)	27.795	0.533565	>0.05
	Prediabetes (n=60)	28.765	0.64721	>0.05
	T2DM (n=60)	28.63167	0.631604	>0.05
Glucose (mg/dL)	Control (n=60)	85.5	1.067364	<0.001
	Prediabetes (n=60)	117.15	0.551744	<0.001
	T2DM (n=60)	208.2667	9.514243	<0.001
HbA1c (%)	Control (n=60)	5.005	0.037161	<0.001
	Prediabetes (n=60)	6.097167	0.028457	<0.001
	T2DM (n=60)	9.09	0.207115	<0.001
TC (mg/dL)	Control (n=60)	177.85	1.543498	<0.001
	Prediabetes (n=60)	216.4167	1.12679	<0.001
	T2DM (n=60)	235.8667	1.145482	<0.001
TG (mg/dL)	Control (n=60)	138.7667	2.626394	<0.001
	Prediabetes (n=60)	214.9333	3.358762	<0.001
	T2DM (n=60)	381.7833	5.905331	<0.001
VLDL (mg/dL)	Control (n=60)	27.75333	0.525279	<0.001
	Prediabetes (n=60)	42.98667	0.671752	<0.001
	T2DM (n=60)	76.35333	1.181451	<0.001
LDL (mg/dL)	Control (n=60)	98.91667	0.705624	<0.001
	Prediabetes (n=60)	131.4667	0.778404	<0.001
	T2DM (n=60)	142.35	1.048762	<0.001
HDL (mg/dL)	Control (n=60)	48.98333	0.765607	<0.001
	Prediabetes (n=60)	42.76667	0.455579	<0.001
	T2DM (n=60)	36.3	0.534832	<0.001

**Figure 1:**
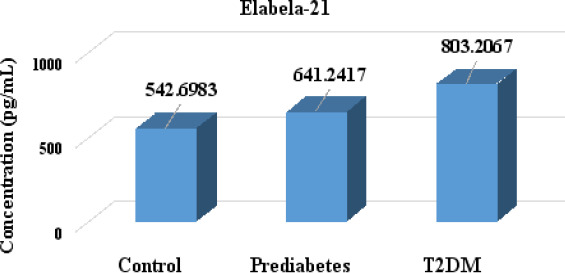
Serum Elabela-21 levels in prediabetes and T2DM patients compared to healthy control

**Figure 2:**
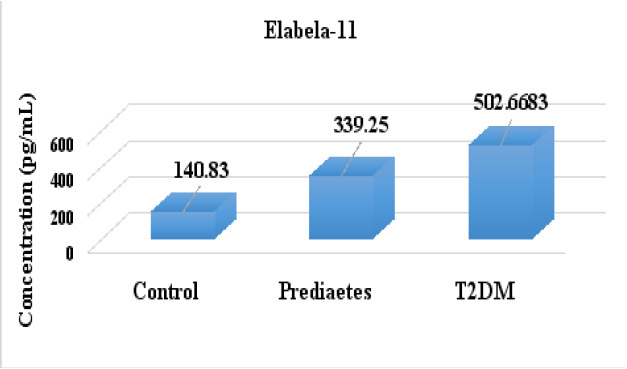
Serum Elabela-11 levels in prediabetes and T2DM patients compared to healthy control

The mean ± SE of serum Elabela-21 in T2DM patients was (803.2067 ± 11.47459 pg/mL), and in prediabetic cases, it was (641.2417 ± 3.216256 pg/mL), which was higher than the mean ± SE value of the healthy controls (542.6983 ± 3.59739 pg/mL), and the differences were statistically significant (P < 0.001) as shown in Figure 1. Also, in Figure 2, the mean ± SE of serum Elabela-11 was significantly higher in people with T2DM and prediabetic cases (502.6683 ± 11.51197 and 339.25 ± 3.452898 pg/mL, P < 0.001), respectively, compared to the healthy controls (140.83 ± 3.603656 pg/mL). These differences were statistically significant (P < 0.001).

Table 2 revealed the circulating levels of Elabela-11 and Elabela-21 by physiological age groups (years). Patients with T2DM and prediabetes in group 3 (61-75 years) had higher levels of Elabela-21 and Elabela-11 compared to healthy controls with statistical variations (P < 0.001), and patients with T2DM and prediabetes in group 2 (46–60 years) had higher Elabela-21 and Elabela-11 values compared to healthy participants with high significant differences (P < 0.001). Likewise, group 1 (30–45 years) had significantly higher circulating concentrations of Elabela-21 and Elabela-11 in cases of newly developed T2DM and prediabetes than in healthy participants (P < 0.001).

**Table 2:** Comparison of study populations using Elabela-11 and Elabela-21 depending on physiological age groups (years)

Parameters	Groups	Group 1 (Mean ± SE)	Group 2 (Mean ± SE)	Group 3 (Mean ± SE)	P-Value
Elabela-21 (pg/mL)	Control (n=60)	534.6 ± 6.5626	542.08 ± 6.6820	547.31 ± 5.5552	P<0.001 (HS)
	Prediabetes (n=60)	644.7684 ± 6.8285	640.83 ± 4.9680	792.27 ± 4.9879	P<0.001 (HS)
	T2DM (n=60)	820.1111 ± 21.1839	792.27 ± 20.2254	793.31 ± 18.2880	P<0.001 (HS)
Elabela-11 (pg/mL)	Control (n=60)	130.2765 ± 5.0884	139.21 ± 6.3076	150.635 ± 6.8245	P<0.001 (HS)
	Prediabetes (n=60)	341.7368 ± 7.1557	339.56 ± 5.3449	338.4 ± 5.5889	P<0.001 (HS)
	T2DM (n=60)	514.9722 ± 20.5882	496.14 ± 21.5948	490.93 ± 17.8131	P<0.001 (HS)

Group 1 (30-45 years), Group 2 (46-60 years), Groups 3 (61-75 years)

Table 3 demonstrated, there were no remarkable differences (P > 0.05) in the concentrations of Elabela-21 between the males of T2DM patients (797.19 ± 14.05 pg/mL), prediabetic cases (638.12 ± 3.76 pg/mL), and health controls (543.68 ± 5.07 pg/mL) in comparison to the females of T2DM patients (809.22 ± 18.58 pg/mL), prediabetic cases (644.36 ± 5.23 pg/mL), and health controls (541.72 ± 5.18 pg/mL). In addition to that, there were no apparent significant differences (P>0.05) in the levels of Elabela-11 between the males of T2DM patients (494.48 ± 13.51 pg/mL), prediabetic cases (335.88 ± 4.30 pg/mL), and health controls (139.65 ± 5.16 pg/mL) compared to the females of T2DM patients (510.86 ± 19.07 pg/mL), prediabetic cases (342.62 ± 5.40 pg/mL), and health controls (142.01 ± 5.11 pg/mL).

**Table 3:** Comparison between study populations based on Elabela-21 and Elabela-11 according to sex

Parameters	Groups	Male (Mean ± SE)	Female (Mean ± SE)	P-Value	C.S.
Elabela-21 (pg/mL)	Control (n=60)	543.68 ± 5.07	541.72 ± 5.18	0.7876	P>0.05 (NS)
	Prediabetes (n=60)	638.12 ± 3.76	644.36 ± 5.23	0.337	P>0.05 (NS)
	T2DM (n=60)	797.19 ± 14.05	809.22 ± 18.58	0.6044	P>0.05 (NS)
Elabela-11 (pg/mL)	Control (n=60)	139.65 ± 5.16	142.01 ± 5.11	0.7464	P>0.05 (NS)
	Prediabetes (n=60)	335.88 ± 4.30	342.62 ± 5.40	0.334	P>0.05 (NS)
	T2DM (n=60)	494.48 ± 13.51	510.86 ± 19.07	0.4817	P>0.05 (NS)

Based on the results in Table 4, there were significant variations (P < 0.001) in the elevated levels of circulating Elabela-21 and Elabela-11 between the obese cases with newly identified T2DM and prediabetes in comparison to the healthy controls. Additionally, there were elevated circulating levels of Elabela-21 and Elabela-11 in the overweight cases with recently developed T2DM and prediabetes more than in healthy controls with highly significant differences (P < 0.001). Furthermore, there were also statistically significant changes (P < 0.001) in Elabela-21 and Elabela-11 levels between normal-weight subjects with newly formed T2DM and prediabetes compared to healthy individuals.

**Table 4:** Comparison between study populations based on Elabela-21 and Elabela-11 according to BMI

Parameters	Groups	Normal weight (Mean ± SE)	Overweight (Mean ± SE)	Obese (Mean ± SE)	P-Value
Elabela-21 (pg/mL)	Control (n=60)	514.595 ± 1.5177	536.075 ± 1.6550	577.425 ± 2.8577	P<0.001 (HS)
	Prediabetes (n=60)	616.86 ± 1.6869	635.905 ± 1.8984	665.3063 ± 3.2628	P<0.001 (HS)
	T2DM (n=60)	723.23 ± 3.3103	768.245 ± 3.0884	910.4944 ± 10.4782	P<0.001 (HS)
Elabela-11 (pg/mL)	Control (n=60)	113.28 ± 1.7722	134.205 ± 1.5700	175.005 ± 3.4123	P<0.001 (HS)
	Prediabetes (n=60)	311.55 ± 1.7139	334.81 ± 1.8443	366.9312 ± 2.9899	P<0.001 (HS)
	T2DM (n=60)	425.74 ± 3.3497	470.12 ± 2.7671	603.15 ± 14.7537	P<0.001 (HS)

Normal weight (18.5 – 24.9 Kg/m^2^), Overweight (25.0 – 29.9 Kg/m^2^), Obese (≥ 30 Kg/m^2^)

Correlation analysis between numerical variables in Table 5 showed no significant associations between BMI with both glucose (0.049304) and HbA1c (0.055903). While BMI had significant weak positive correlations with circulating levels of TC (0.237091), TG (0.313951), and LDL-C (0.232645). Whereas a significant weak negative association between BMI and HDL-C (-0.31131).

**Table 5:** Correlation analysis between numerical variables

Variables	BMI	Glucose	HbA1c	TC	TG	LDL	HDL	Elabela-21	Elabela-11
**BMI**	1	0.049304	0.055903	0.237091	0.313951	0.232645	-0.31131	0.429577	0.362002
**Glucose**	0.049304	1	0.831815	0.639822	0.732732	0.627229	-0.5738	0.682436	0.679505
**HbA1c**	0.055903	0.831815	1	0.743024	0.834775	0.711673	-0.63354	0.757691	0.765397
**TC**	0.237091	0.639822	0.743024	1	0.84713	0.941022	-0.56167	0.828086	0.900786
**TG**	0.313951	0.732732	0.834775	0.84713	1	0.836005	-0.7856	0.966339	0.950877
**LDL**	0.232645	0.627229	0.711673	0.941022	0.836005	1	-0.66133	0.848006	0.921096
**HDL**	-0.31131	-0.5738	-0.63354	-0.56167	-0.7856	-0.66133	1	-0.76501	-0.77173
**Elabela-21**	0.429577	0.682436	0.757691	0.828086	0.966339	0.848006	-0.76501	1	0.972395
**Elabela-11**	0.362002	0.679505	0.765397	0.900786	0.950877	0.921096	-0.77173	0.972395	1

Glucose had significant, strong associations with HbA1c (0.831815) and TG (0.732732); in addition, glucose had significant, moderate correlations with TC (0.639822) and LDL-C (0.627229). On the contrary, a significant moderate inverse relationship between glucose and HDL-C (-0.5738).

Significant positive strong correlations were found between HbA1c and serum concentrations of TC (0.743024), TG (0.834775), and LDL-C (0.711673). While, there was a significant positive moderate relationship between HbA1c and HDL-C (-0.63354).

Serum TC levels had significant strong positive associations with triglycerides (0.84713) and LDL-C (0.941022). Whereas serum TC levels had a significant moderate negative relationship with HDL-C (-0.56167). A significant strong positive correlation existed between TG and LDL-C (0.836005). Conversely, TG had a significant strong negative association with HDL-C (-0.7856). A significant opposite, moderate correlation was found between circulating levels of LDL-C and HDL-C (-0.66133). Serum Elabela-21 levels were significantly and moderately positively correlated with BMI (0.429577) and glucose (0.682436) and significantly and strongly positively associated with levels of HbA1c (0.757691), TC (0.828086), TG (0.966339), and LDL-C (0.848006). On the contrary, there is a significant negative strong correlation between Elabela-21 and HDL-C (-0.76501).

Serum Elabela-11 levels had a significantly positive weak correlation with BMI (0.362002) and a significantly positive moderate association with glucose (0.679505) and significantly strong positive relationships with levels of HbA1c (0.765397), TC (0.900786), TG (0.950877), LDL-C (0.921096), and Elabela-21 (0.972395). Otherwise, a significant negative strong correlation between Elabela-11 and HDL-C (-0.77173).

## Discussion

The Elabela gene encodes a 54-amino-acid pre-proElabela with a signal peptide, which is translocated into the endoplasmic reticulum and cleaved into proElabela of 32 amino acids and then cleaved into active fragments ELA-21 and ELA-11^18^.

This study demonstrated that circulating Elabela-21 and Elabela-11 levels progressively increased from prediabetes to recently developed type 2 diabetes. Therefore, these results may display changes in the concentrations of Elabela-21 and Elabela-11 over time. A recent study^19^ found that serum Elabela levels were considerably lower in patients with T2DM than in healthy people. It considered the possibility of a connection between lower Elabela levels and T2DM complications.

Onalan et al., found that Elabela values are considerably reduced in individuals with T2DM than in healthy people. They gradually drop from no albuminuria to macroalbuminuria, suggesting that it may be a biomarker for the severity of diabetic nephropathy^20^.

This disagreement may be a result of our study cases recently having T2DM which may decrease the levels of Elabela-21 and Elabela-11 progressively with time in T2DM complications.

The Apelin/APJ system is highly generated in human tissues and controls several vital biological processes, such as insulin secretion and fluid homeostasis^21^. Elabela-21 and Elabela-11 hormones may play an important role in the pathogenesis of T2DM and insulin resistance. Although the current study did not allow us to deduce the cause of Elabela-21 and Elabela-11 levels gradually increasing from prediabetes to T2DM, the study suggests these results might be a defensive response to metabolic stress from resistance to Elabela-21 and Elabela-11 action. The increased concentration of Elabela-21 and Elabela-11 in prediabetes and newly identified T2DM patients may be the result of the impairment of Elabela-21 and Elabela-11 signaling in specific tissues and the dysregulation of Elabela-21 and Elabela-11 synthesis or a response to hyperglycemia and hyperinsulinemia in the T2DM state.

Circulating Elabela-21 and Elabela-11 were positively correlated with BMI, glucose, HbA1c, TC, TG, and LDL-C. On the contrary, they were negatively associated with HDL. It's probable that insulin or glucose regulate the levels of Elabela-21 and Elabela-11 in the blood. Elabela can function through APJ and activates downstream signaling pathways, involving mitogen-activated protein kinase 3 (MAPK3/1) and PI3K/AKT/mammalian target of rapamycin kinase (mTOR). It also has a function in the regulation of a several of processes, such as food intake^22^,^23^. The signaling cascade that is mainly activated by Elabela-APLNR interaction has been identified as the PI3K-AKT pathway. P3 is a crucial intracellular second messenger that is necessary for protein kinase B (AKT) to be transferred to the membrane for activation^24^,^25^. Through a variety of channels, insulin and other growth hormones mediate the phosphorylation of AKT, which stimulates cell proliferation and enhances cell survival. A different pathway that is stimulated is ERK/MAPK, which is triggered via ELA-11^26^.

Yeniel et al. found no association between Elabela levels and glucose, insulin, or lipid profile in obese individuals who do not have T2DM and other chronic diseases^11^. This could indicate that the levels of Elabela-21 and Elabela-11 rise, specifically in obese individuals with T2DM, is a difference in the number of participants and total Elabela level measured. The study's main limitations are the small number of participants, its focus on the Basra population, and the detection of only Elabela-21 and Elabela-11 isoforms.

## Conclusion

This study demonstrated that levels of the hormones Elabela-21 and Elabel-11 in the blood gradually increased from prediabetes to T2DM. They were positively associated with BMI, glucose, HbA1c, TC, TG, and LDL-C are inversely associated with HDL. Accordingly, Elabela-21 and Elabel-11 may be helpful predictive biomarkers in diagnosing prediabetes and T2DM. Nevertheless, additional studies are required to elucidate the biological process involving Elabela-21 and Elabel-11 in the etiology of T2DM.
